# Association of Intraoperative Parathyroid Hormone Decline with Early Postoperative Hypocalcemia: A Single-Center Retrospective Study

**DOI:** 10.3390/diagnostics16040636

**Published:** 2026-02-22

**Authors:** Suat Evirgen, Elif Menekse, Ecem Avci, Burak Yasin Avci, Çiğdem Tura Bahadır, Cafer Polat

**Affiliations:** 1 Department of General Surgery, Serefeddin Sabuncuoğlu Training and Research Hospital, Amasya University, 05200 Amasya, Türkiye; 2 Department of Medical Biochemistry, Serefeddin Sabuncuoğlu Training and Research Hospital, Amasya University, 05200 Amasya, Türkiye; 3 Division of Endocrinology, TOBB ETU Hospital, 06510 Ankara, Türkiye

**Keywords:** primary hyperparathyroidism, parathyroid tumor, intraoperative PTH, alkaline phosphatase, ROC analysis, diagnostic accuracy, Youden index

## Abstract

**Background/Objectives**: Postoperative early hypocalcemia (PEH) is a key postoperative issue after parathyroidectomy in primary hyperparathyroidism. It often leads to long-lasting hypocalcemia, requiring more calcium and active vitamin D supplements. This study aimed to determine whether the extent of intraoperative parathyroid hormone (PTH) decline, measured 15 min after parathyroid tumor excision, could serve as a reliable intraoperative rule-out marker for PEH. **Methods**: We conducted a retrospective review of 88 adult patients who underwent surgical intervention for a solitary parathyroid tumor at a single institution. Postoperative early hypocalcemia (PEH) was defined as a total serum calcium level <8.5 mg/dL within the postoperative 6th hour or on postoperative day 1, requiring clinical calcium supplementation (oral and/or intravenous), with active vitamin D when appropriate. The percentage decrease in PTH at 15 min post-excision was calculated using morning-of-surgery preoperative PTH values alongside the 15-min post-excision levels. Additional variables assessed included preoperative alkaline phosphatase (ALP), parathyroid tumor weight, and serum concentrations of calcium, phosphate, magnesium, and 25-hydroxyvitamin D. Predictive factors were identified by logistic regression, and the diagnostic accuracy of the 15-min PTH decline was evaluated using receiver operating characteristic (ROC) curve analysis, optimizing cutoff selection with Youden’s index. Odds ratios were standardized per 10-unit increments for ALP and parathyroid tumor weight for interpretability. **Results**: Of the studied cohort, 10 patients (11.4%) developed PEH. The intraoperative 15-min PTH decline was notably greater in those who developed PEH compared to those who did not (81.2 ± 4.4% vs. 69.9 ± 8.3%; *p* < 0.001). Univariate logistic regression showed a significant association between the 15-min PTH decline and PEH (OR 1.22 per 1% increment; 95% CI 1.08–1.38). That said, when we added ALP and parathyroid tumor weight to the multivariate models, PTH decline no longer predicted independently. In contrast, ALP (OR 3.11 per 10 U/L; 95% CI 1.34–7.93; *p* = 0.011) and parathyroid tumor weight (OR 1.22 per 10 mg; 95% CI 1.10–1.48; *p* = 0.004) stayed significant. Thus, the incremental prognostic contribution of the 15-min PTH decline beyond ALP and parathyroid tumor weight appears limited. The ROC curve for the 15-min PTH decline produced an AUC of 0.883, with an optimal cutoff of 75% providing 100% sensitivity and 74.4% specificity. No patients with a PTH decline below 75% developed PEH. **Conclusions**: Preoperative ALP and parathyroid tumor weight showed the strongest independent associations with PEH following parathyroid tumor surgery. An intraoperative PTH decline of less than 75% at 15 min may serve as a practical rule-out tool for PEH, although further validation in larger patient populations is warranted.

## 1. Introduction

Primary hyperparathyroidism (PHPT) is recognized as one of the most prevalent endocrine diseases, with a solitary parathyroid tumor constituting its most frequent etiology. Surgical excision stands as the definitive treatment in correctly selected patients [1]. In contemporary surgical protocols, intraoperative monitoring of parathyroid hormone (ioPTH) levels has been embraced as an important adjunct, providing real-time biochemical feedback to guide operative decision-making and, when appropriately applied, minimizing the need for unnecessary bilateral neck exploration [2]. However, substantial variability exists in published protocols regarding ioPTH criteria and sampling methodology, and numerous cutoff algorithms have been proposed to optimize intraoperative judgment. This underlines the need to better define how early PTH kinetics can be translated into outcomes that hold meaningful clinical utility beyond immediate surgical success [3].

Early postoperative hypocalcemia (PEH) remains a major postoperative concern. It shows up as ongoing hypocalcemia after parathyroidectomy, caused by quick bone remineralization in a long-term demineralized skeleton. Patients usually need a lot of calcium and active vitamin D, plus careful monitoring of blood levels [4]. Incidence and severity of PEH range considerably across different studies and healthcare settings, likely reflecting variations in baseline skeletal disease burden, biochemical phenotype, and perioperative management strategies [5].

Multiple preoperative markers—most notably, elevated alkaline phosphatase (ALP) levels—have consistently been linked with a higher likelihood of postoperative hypocalcemia and PEH. These markers can help identify patients who may benefit from anticipatory supplementation and close monitoring in the perioperative period [6]. Strategies such as vitamin D optimization before surgery have also been explored as possible interventions to minimize the clinical impact of hypocalcemia and decrease the likelihood of PEH in select patients [7].

Evolving clinical research continues to highlight the multifactorial nature of the likelihood of PEH. The degree of preoperative biochemical impairment and evidence of increased bone turnover seem particularly instructive, supporting the importance of multivariable risk assessment over reliance on any single parameter or marker [8]. At the same time, the utility of straightforward preoperative tests—such as ALP-based thresholds—for risk stratification and management planning remains a focus in studies seeking practical solutions for diverse clinical contexts [9]. Larger, contemporary series from differently resourced settings demonstrate both the practical burden of PEH and the need for implementable predictors to inform perioperative care [10].

Within this context, our retrospective diagnostic accuracy study sought to evaluate whether the magnitude of the parathyroid hormone decline 15 min after parathyroid tumor excision could effectively serve as an intraoperative rule-out test for PEH following surgery for solitary parathyroid tumor. Furthermore, we aimed to characterize additional preoperative predictors relevant for everyday clinical practice that could assist in risk stratification and patient management.

In routine practice, this situation often leads to longer in-hospital observation, more frequent biochemical follow-up, and, in some cases, unplanned reassessment or readmission due to symptomatic hypocalcemia. Therefore, identifying low-risk patients as early as possible—ideally during the intraoperative period—is crucial for both patient safety and efficient use of hospital resources.

## 2. Materials and Methods

### 2.1. Study Design and Ethical Approval

This retrospective diagnostic accuracy investigation was conducted at the Department of General Surgery, Amasya University Serefeddin Sabuncuoğlu Training and Research Hospital. Institutional ethical approval was secured from the local ethics board (Approval number: 258140, Approval date: 28 April 2025), and the study adhered to the principles set forth in the Declaration of Helsinki. All identifiable patient information was de-identified prior to analysis, and all data handling and reporting were performed on anonymized records.

### 2.2. Patient Selection

Eligible participants included consecutive adults who underwent surgical intervention for primary hyperparathyroidism attributed to a solitary parathyroid tumor between January 2019 and February 2025. A total of 88 patients met the inclusion criteria and comprised the final study cohort. All patients were followed through the immediate postoperative period, with biochemical assessments performed at 6 h and 24 h post-surgery, and clinical follow-up until hospital discharge.

Inclusion criteria were as follows: (1) age ≥18 years; (2) biochemical diagnosis of primary hyperparathyroidism with elevated serum PTH and calcium; (3) preoperative imaging (cervical ultrasonography and/or scintigraphy) consistent with a solitary parathyroid tumor; (4) surgical and histopathological confirmation of a solitary parathyroid tumor; (5) availability of complete preoperative biochemical data (calcium, phosphate, magnesium, PTH, ALP, and 25-hydroxyvitamin D); (6) availability of intraoperative PTH measurements at baseline and 15 min post-excision; and (7) availability of postoperative biochemical surveillance (calcium at 6 h and day 1).

Exclusion criteria included: (1) multiple endocrine neoplasia syndromes or familial hyperparathyroidism; (2) secondary or tertiary hyperparathyroidism related to chronic kidney disease (eGFR <60 mL/min/1.73 m^2^ or dialysis); (3) prior neck surgery; (4) multigland disease or parathyroid hyperplasia; (5) incomplete laboratory data; and (6) concurrent malignancy or severe systemic illness potentially affecting calcium metabolism.

This careful screening ensured the study population represented a homogeneous sample with complete biochemical and pathological evaluation.

### 2.3. Surgical Approach and Parathyroid Hormone Measurement

Preoperative localization was routinely performed using a combination of cervical ultrasonography and/or scintigraphy in accordance with institutional protocol. The surgical strategy was planned as either a focused minimally invasive approach or classical bilateral neck exploration, based on imaging findings and the surgeon’s clinical judgment.

Serum parathyroid hormone (PTH) measurements were obtained at two predefined time points:

**Preoperative baseline PTH:** In our institution, the preoperative baseline PTH was measured from a venous blood sample drawn on the morning of surgery, in line with our routine protocol. Because the time between diagnosis and surgery can often span weeks to months—and PTH is not routinely rechecked during this waiting period—using a contemporaneous baseline provides a current reference for interpreting intraoperative kinetics and calculating the percent decline. No additional baseline PTH sampling was performed for research purposes; the measurement was part of routine preoperative blood work. Although some centers obtain a comparable baseline value within 24 h before surgery, we routinely collect it on the morning of the procedure to keep the timing close to the intraoperative samples and to maintain consistency in sampling and measurement.

**Post-excision 15-min PTH:** Measured from a venous blood sample obtained exactly 15 min after parathyroid tumor removal.

The proportional decline at 15 min was calculated as follows:

15-min PTH decline (%) = (Baseline PTH − 15-min PTH)/Baseline PTH × 100

When available, postoperative day 1 PTH was also recorded, and the percent change relative to baseline was reported as a secondary biochemical metric.

### 2.4. Assessment of Biochemical and Pathological Variables

Preoperative laboratory parameters evaluated included total serum calcium, phosphate, magnesium, 25-hydroxyvitamin D, and alkaline phosphatase (ALP). Postoperative biochemical surveillance entailed measurement of serum calcium at 6 h post-surgery and on the first postoperative day; phosphate and magnesium were rechecked on day one postoperatively.

The following indices were included in the analytic plan:

15-min PTH decline (%)
Postoperative day 1 PTH decline (%)
Percentage change in serum calcium at 6 h and on postoperative day 1 compared to preoperative levels
Preoperative ALP concentration
Parathyroid tumor size and weight (documented from histopathology reports)

### 2.5. Definition of Postoperative Early Hypocalcemia (PEH)

The primary clinical endpoint of this study, postoperative early hypocalcemia (PEH), was defined as the documentation of hypocalcemia in the medical records during the early postoperative period (within the postoperative 6th hour and first postoperative day evaluations), i.e., a total serum calcium level <8.5 mg/dL, requiring clinical treatment. Accordingly, cases requiring high-dose oral calcium and/or intravenous calcium supplementation were considered within the scope of PEH, and the use of active vitamin D analogs (e.g., calcitriol) was also recorded when deemed appropriate. Clinical symptoms associated with hypocalcemia, such as paresthesia, muscle cramps, or tetany, were additionally noted, and accompanying biochemical abnormalities (e.g., phosphate or magnesium disturbances) were documented when available. Patients fulfilling these clinical and laboratory criteria were classified as PEH-positive for the purposes of analysis, while all remaining patients were considered PEH-negative.

### 2.6. Statistical Analysis

All statistical evaluations were carried out using IBM SPSS Statistics, version 26.0 (IBM Corp., Armonk, NY, USA). Continuous variables were summarized as mean ± standard deviation or, where appropriate, as median with minimum and maximum bounds, according to their distribution assessed by the Shapiro–Wilk test and visual examination. Categorical data were reported as absolute numbers and corresponding percentages. Comparative analyses between groups were conducted using independent-samples *t*-tests or Mann–Whitney U-tests for continuous data, and either the chi-square or Fisher’s exact test for categorical variables, depending on data characteristics.

Univariate binary logistic regression models were used to estimate the odds ratios (ORs) and 95% confidence intervals (CIs) for potential predictors of PEH. For interpretational clarity, results for continuous predictors were expressed per relevant incremental unit (1% for the 15-min PTH decline, 10 U/L for ALP, 10 mg for parathyroid tumor weight). Owing to the limited number of PEH events, multivariable models were constrained in complexity to mitigate overfitting. The discriminative performance of the 15-min PTH decline was determined by ROC curve analysis, with the optimal threshold specified by Youden’s index. Detailed metrics including sensitivity, specificity, positive predictive value, and negative predictive value at the identified cutoff were reported. A two-sided *p*-value < 0.05 was considered the threshold for statistical significance throughout all analyses.

## 3. Results

### 3.1. Patient Characteristics

A total of 88 adult patients who underwent surgery for a solitary parathyroid tumor were included in the final analysis. Among these, postoperative early hypocalcemia developed in 10 individuals, representing an incidence of 11.4%, while 78 patients (88.6%) did not experience this complication. Detailed baseline characteristics are provided in Table 1.

There were no statistically significant differences in age or sex distribution between those who developed PEH and those who did not (*p* = 0.99 and *p* = 0.10, respectively). Notably, the mean preoperative serum calcium level was significantly lower in the group that developed PEH compared to those who did not (10.64 ± 0.23 vs. 11.89 ± 0.68 mg/dL; *p* < 0.001). Preoperative levels of parathyroid hormone and 25-hydroxyvitamin D did not differ meaningfully between groups (*p* = 0.56 and *p* = 0.75, respectively). By contrast, preoperative alkaline phosphatase (ALP) levels were substantially elevated in the PEH group (219.6 ± 10.5 vs. 152.1 ± 18.0 U/L; *p* < 0.001), and parathyroid tumor weight was also significantly greater among those who developed PEH (588.4 ± 38.4 vs. 458.9 ± 58.2 mg; *p* < 0.001).

### 3.2. Intraoperative and Early Postoperative Biochemical Changes

Table 2 summarizes perioperative biochemical trajectories. The percentage decline in PTH at 15 min intraoperatively was notably higher in patients developing PEH compared to non-PEH patients (81.2 ± 4.4% vs. 69.9 ± 8.3%; *p* < 0.001). Similarly, the percentage reduction in PTH on postoperative day 1 was more pronounced in the PEH group (85.5 ± 4.6% vs. 75.9 ± 7.1%; *p* < 0.001).

With respect to serum calcium, patients who developed PEH demonstrated a significantly greater mean percentage decrease at both 6 h postoperatively and on the first post-op day (6-h: 27.0 ± 3.0% vs. 20.8 ± 4.5%; day 1: 34.2 ± 2.8% vs. 28.0 ± 4.2%; both *p* < 0.001).

### 3.3. Predictors of Postoperative Early Hypocalcemia: Logistic Regression Analysis

Table 3 presents the results of logistic regression analyses for predictors of PEH. In univariate analysis, the 15-min PTH decline magnitude showed a significant association with the likelihood of developing PEH (OR 1.22 per 1% increase; 95% CI 1.08–1.38; *p* = 0.001). Both preoperative ALP and parathyroid tumor weight demonstrated strong univariate associations with PEH (*p* = 0.003 and *p* < 0.001, respectively).

However, in multivariable models that adjusted for possible confounding between predictors, the 15-min PTH decline did not retain independent significance when ALP (*p* = 0.91) or parathyroid tumor weight (*p* = 0.11) were included in the model. Both ALP (adjusted OR 3.11 per 10 U/L increment; 95% CI 1.34–7.93; *p* = 0.011) and parathyroid tumor weight (adjusted OR 1.22 per 10 mg increment; 95% CI 1.10–1.48; *p* = 0.004) remained independently associated. Accordingly, given its limited incremental prognostic contribution beyond ALP and parathyroid tumor weight, the ROC-based cutoff for the 15-min PTH decline is presented as a practical intraoperative rule-out tool for PEH (discrimination), rather than as an independent predictor in adjusted multivariable models.

### 3.4. Diagnostic Utility of the 15-Min PTH Decline

**Figure 1:**
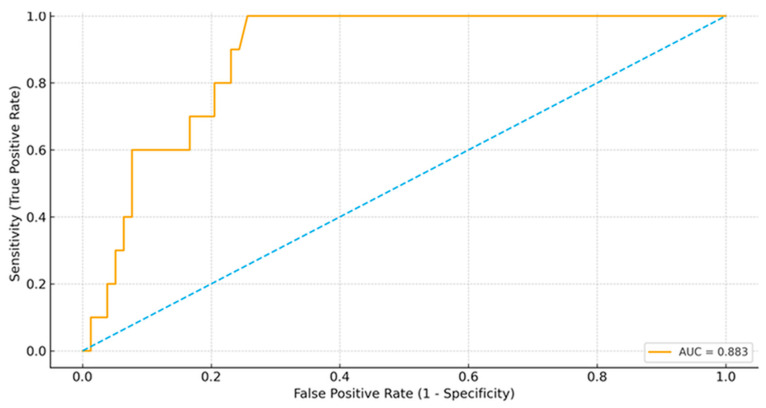
Receiver operating characteristic (ROC) curve of the 15-min intraoperative PTH decline for predicting postoperative early hypocalcemia after parathyroid tumor excision. The area under the curve (AUC) is 0.883, indicating good diagnostic accuracy. The diagonal line represents the reference line (AUC = 0.5).

Evaluation of the diagnostic accuracy of the 15-min PTH decline using ROC analysis demonstrated robust performance for predicting PEH (AUC = 0.883; see Table 4 and Figure 1). ROC analysis evaluates discriminatory performance rather than independent predictive effects; thus, this cutoff is intended for intraoperative rule-out use. The optimal cutoff, as determined by maximization of the Youden index, was a 75% decline. This threshold corresponded to a sensitivity of 100% and a specificity of 74.4%, yielding a positive predictive value (PPV) of 33.3%, a negative predictive value (NPV) of 100%, and an overall diagnostic accuracy of 77.3%.

**Figure 2:**
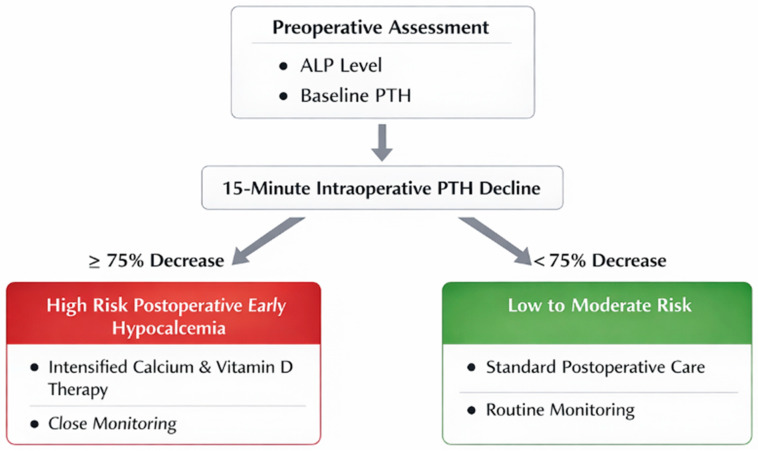
It presents a practical decision pathway for assessing the risk of postoperative early hypocalcemia (PEH) after parathyroid tumor surgery. Preoperative ALP and baseline PTH are evaluated, and the percentage decline in PTH is calculated 15 min after parathyroid tumor excision. A decline of ≥75% points to a higher-risk profile for PEH and may support considering closer monitoring and intensified calcium/active vitamin D supplementation. In contrast, a decline of <75% suggests a low-to-moderate risk profile and supports a PEH “rule-out” approach, favoring standard postoperative care and routine monitoring.

Of particular note, no cases of PEH were observed among patients with a 15-min PTH decline of less than 75%. Figure 2 provides a simplified risk stratification model integrating 15-min PTH decline with preoperative ALP.

## 4. Discussion

In this retrospective cohort of 88 patients who underwent surgery for a parathyroid tumor, postoperative early hypocalcemia (PEH) occurred in 10 patients (11.4%). This rate is comparable to contemporary series in primary hyperparathyroidism (PHPT), where PEH can prolong hospitalization and necessitate calcium and active vitamin D replacement despite successful parathyroidectomy [6,11,12]. Our data showed no differences in age or sex between patients with and without PEH, suggesting that early postoperative calcium changes are more closely related to disease biology and skeletal turnover than to basic demographic factors [6,11,12].

One notable finding was that patients who developed PEH had a distinct preoperative biochemical profile. Mean preoperative serum calcium was significantly lower in the PEH group than in those who remained normocalcemic postoperatively. In PHPT, a relatively lower baseline calcium can be seen in patients with more pronounced skeletal involvement and high bone turnover, where calcium is continuously utilized for remineralization. After parathyroid tumor excision, the abrupt reduction in PTH-driven bone resorption, together with ongoing skeletal mineral uptake, may lead to a rapid net shift of calcium into bone and result in early postoperative hypocalcemia [6,13,14,15]. Thus, lower baseline calcium in PHPT should not be interpreted as indicating milder disease; rather, in the appropriate clinical context, it may reflect advanced skeletal involvement and an association with postoperative calcium derangement.

In our study, among preoperative markers, alkaline phosphatase (ALP) stood out as the variable most strongly associated with PEH. Preoperative alkaline phosphatase is a practical marker of bone turnover, primarily reflecting osteoblastic activity driven by long-standing PTH exposure. ALP levels were higher in patients with PEH and remained independently associated with PEH after multivariable adjustment (OR 3.11 per 10 U/L increase; 95% CI, 1.34–7.93; *p* = 0.011). This fits with the idea that PEH comes from fast bone repair after surgery. It also aligns with multiple studies that have emphasized bone turnover markers as clinically actionable indicators for anticipating postoperative calcium requirements [6,13,14,15].

Parathyroid tumor weight also showed a significant relationship with PEH in our series and remained independently associated in multivariable analysis (OR 1.22 per 10 mg increment, 95% CI 1.10–1.48; *p* = 0.004). This suggests that tumor burden correlates meaningfully with postoperative calcium kinetics. Larger parathyroid tumors likely reflect longer disease duration or more intense hormonal secretion—or both—resulting in greater cumulative PTH exposure and more pronounced skeletal consequences. After excision, the greater the extent of skeletal depletion, the more vigorous the remineralization response, translating into greater calcium uptake and increased likelihood of PEH [6,16].

By contrast, baseline preoperative PTH concentration did not discriminate between patients with and without PEH in our cohort. This observation is consistent with clinical experience showing that a single preoperative PTH value can be highly variable, is subject to assay-specific factors, and does not always correlate well with the extent of skeletal involvement. Consequently, preoperative PTH alone often proves to be an unreliable stand-alone predictor of postoperative hypocalcemia or PEH in PHPT [11,17,18,19].Our findings thus support prioritizing markers that reflect cumulative skeletal impact—such as ALP—and disease burden—such as parathyroid tumor weight—rather than relying exclusively on a single hormone measurement.

Although vitamin D status is considered a potentially modifiable factor in relation to postoperative hypocalcemia, 25-hydroxyvitamin D levels were comparable between patients with postoperative early hypocalcemia and those without, likely reflecting the high background prevalence of vitamin D deficiency that limits baseline variability. Moreover, many clinics now give vitamin D before surgery, which could even out differences by operation time. In such settings, a single preoperative 25(OH)D measurement may lose discriminative power even though vitamin D optimization remains clinically important and may reduce the severity of postoperative hypocalcemia [6,7]. It is important to emphasize that the lack of difference in 25(OH)vitamin D in our series should not be misconstrued as evidence that vitamin D is irrelevant. Rather, it suggests that in our clinical pathway, vitamin D may function more as a background modifier than as a primary discriminator. Given the limited number of PEH events, our study may have been underpowered to detect a modest association between 25(OH) vitamin D and PEH. Therefore, this negative finding should not be interpreted as contradicting the well-established relationship between vitamin D status and postoperative hypocalcemia reported in prior literature.

Postoperative calcium kinetics in our data further supported a dynamic interpretation of PEH. Patients who developed PEH exhibited a steeper early decline in serum calcium—both at 6 h and on postoperative day 1—consistent with rapid skeletal uptake of mineral. This pattern fits well with the typical clinical course wherein clinically significant hypocalcemia often becomes apparent within the first 48 to 72 h following successful parathyroidectomy [11,12,13,20]. From a practical standpoint, monitoring early calcium trajectories—not merely nadir values—may help clinicians anticipate which patients will require aggressive supplementation and more intensive biochemical surveillance. Such early signals may be particularly valuable in settings where same-day or short-stay discharge pathways are being considered.

A clinically actionable contribution of this study is the evaluation of intraoperative PTH kinetics as a procedure-integrated tool for risk stratification. While ioPTH monitoring is traditionally employed to confirm adequate biochemical response and guide operative strategy, our findings suggest that the magnitude of the PTH decline also conveys prognostic information about postoperative calcium metabolism. In our cohort, all cases of PEH occurred among patients who had a 15-min PTH decline of 75% or greater, whereas no patient with a decline below 75% developed PEH. This yielded the following performance: sensitivity 100%, specificity 74.4%, positive predictive value 33.3%, negative predictive value 100%, and an AUC of 0.883 (95% CI 0.815–0.951; *p* < 0.001). Taken together, these results support the practical interpretation that a 15-min PTH decline of less than 75% may function effectively as an intraoperative “rule-out” criterion for PEH in our clinical setting. Notably, although the 15-min PTH decline did not remain independently significant in adjusted models, its high NPV supports its use as a practical intraoperative rule-out marker. However, because ALP and parathyroid tumor weight showed stronger and more consistent independent associations with PEH, the 15-min PTH decline should be viewed primarily as a complementary intraoperative rule-out/triage parameter rather than a stand-alone prognostic marker.

It is important to note, however, that the 75% threshold should not be regarded as a definitive diagnostic test for PEH. A substantial proportion of patients without PEH also exhibited 15-min PTH declines of 75% or more, which limits the positive predictive value. Therefore, the most appropriate clinical application of this marker is for triage rather than confirmation. Importantly, this approach is intended for centers where perioperative PTH testing is already embedded in routine workflows and should not be taken as a rationale to add extra preoperative PTH measurements purely for PEH prediction. A PTH decline below 75% supports de-escalation of postoperative monitoring intensity in otherwise stable patients, whereas a decline at or above 75% should prompt integration with other preoperative indicators of skeletal turnover and disease burden. In an integrated risk assessment model, a patient presenting with elevated ALP, a large parathyroid tumor, and a marked 15-min PTH fall would be considered at higher likelihood of PEH and might benefit from early prophylactic calcium and active vitamin D strategies along with more intensive biochemical follow-up. Conversely, a patient with normal ALP and a smaller parathyroid tumor may be managed with standard postoperative care even if a substantial ioPTH drop is observed [21,22].

Beyond its well-known effects on bone and renal calcium handling, sustained PTH excess may also influence systemic metabolism. Modica and colleagues recently reviewed evidence linking parathyroid disorders to components of the metabolic syndrome, including adverse cardiometabolic profiles and insulin resistance. This broader metabolic context may be worth keeping in mind when interpreting perioperative biochemical changes in PHPT [23].

To make these results easier to use in daily practice, we suggest a simple clinical decision algorithm that combines preoperative ALP with the intraoperative 15-min PTH decline to support early postoperative monitoring and supplementation decisions (Figure 2). In this approach, a 15-min PTH decline of less than 75% is mainly used as a practical intraoperative rule-out indicator for PEH and may support standard postoperative care in otherwise stable patients. In contrast, a decline of 75% or more—especially when ALP is elevated—may point to patients who could benefit from closer biochemical follow-up and earlier, more intensive calcium and active vitamin D replacement.

In addition to the limitations detailed below, several endocrinology-focused aspects should be noted. First, calcium assessment in this study was based on total serum calcium; ionized calcium was not consistently available, and albumin-corrected calcium was not calculated due to the retrospective design. Second, a standardized postoperative endocrine subclassification (e.g., hypocalcemia with normal PTH versus hypocalcemia due to hypoparathyroidism, or a dedicated hungry bone syndrome categorization) could not be performed because uniform longitudinal calcium–PTH follow-up and a comprehensive bone turnover marker panel were not routinely available. In this context, ALP was interpreted as an indirect marker of bone metabolic activity, while other markers (e.g., P1NP, CTX, osteocalcin) were not available for systematic analysis.

In accordance with the 2022 WHO classification and its updated nomenclature for parathyroid neoplasia, we used the umbrella term “parathyroid tumor” throughout the manuscript. The updated WHO scheme includes relevant nomenclature changes (e.g., replacement of “atypical parathyroid adenoma” with “atypical parathyroid tumor”), reflecting current consensus terminology. Therefore, the term “parathyroid tumor” was preferred in the general description of the study population, while specific histopathological diagnoses were reported as documented in pathology records [24].

This study has several strengths, including the evaluation of a relatively homogeneous cohort restricted to solitary parathyroid tumors, the use of standardized timing for intraoperative PTH measurements, and the systematic assessment of early postoperative serum calcium kinetics at consecutive time points. In addition, considering a routinely available marker such as ALP alongside intraoperative PTH dynamics enhances the clinical interpretability and potential applicability of our findings. Methodological limitations and issues related to generalizability are addressed in detail in the Limitations section below.

### Limitations

Several limitations warrant acknowledgment. First, the retrospective single-center design limits generalizability and introduces the possibility of unmeasured confounding variables. Because systematic genetic testing was not available for all patients, we excluded individuals with documented or clinically suspected hereditary hyperparathyroidism (e.g., MEN) based on medical records and family history; however, unrecognized genetic forms cannot be completely ruled out. Second, the relatively modest number of PEH events (*n* = 10) constrains the complexity of multivariable models and may reduce the precision of effect estimates. This limited event count also reduces the ability to detect potentially relevant associations for secondary variables such as 25(OH) vitamin D. Because surgery for solitary parathyroid tumor is relatively infrequent in routine practice and PEH occurs only in a minority of cases, our cohort was limited to consecutive patients with complete data over the study period, which inevitably resulted in a modest sample size and event count. Third, bone mineral density measurements, standardized radiographic assessments of skeletal involvement, and extended panels of bone turnover markers were not uniformly available in our cohort, precluding more detailed phenotypic characterization of skeletal disease. Furthermore, the validity of the proposed threshold values in specific subgroups, such as patients with renal impairment, who were excluded from this study, remains to be determined. Finally, external validation in independent, multicenter cohorts is essential before adopting any specific intraoperative PTH threshold as a broadly applicable clinical standard. In addition, we did not perform a formal cost-effectiveness analysis; therefore, our findings should not be interpreted as recommending additional baseline PTH testing solely to predict PEH in settings where such testing is not already part of routine perioperative care.

A post-hoc power analysis indicated approximately 80% power for detecting the primary association between PTH decline and PEH, given the sample size.

## 5. Conclusions

In this study, we demonstrated that the intraoperative 15-min parathyroid hormone decline percentage provides valuable prognostic information that extends beyond its traditional role in confirming operative success. While elevated preoperative alkaline phosphatase and increased parathyroid tumor weight emerged as the strongest independent predictors of postoperative early hypocalcemia (PEH), the intraoperative 15-min PTH decline did not remain independently significant in adjusted models; nevertheless, it provides a practical and reliable intraoperative rule-out test. Specifically, a decline of less than 75% at 15 min reliably excluded the subsequent development of PEH in our cohort, achieving % 100 sensitivity and strong negative predictive value.

From a clinical perspective, this suggests that patients exhibiting a PTH decline below this threshold may be considered for standard postoperative care pathways or potentially earlier discharge protocols, whereas those with a more pronounced decline—particularly when accompanied by elevated alkaline phosphatase—warrant closer surveillance and proactive calcium management strategies. Future multicenter prospective studies are needed to validate this 75% threshold in diverse populations and clinical settings, with the ultimate goal of establishing standardized, risk-stratified discharge and monitoring protocols that optimize both patient safety and resource utilization.

## Figures and Tables

**Table 1:** Baseline characteristics of the study population.

Characteristic	PEH (−) (*n* = 78)	PEH (+) (*n* = 10)	*p*-Value
Age (years)	62.1 ± 11.7	62.2 ± 12.0	0.99
Sex (female/male)	65/13	6/4	0.10 ^a^
Preoperative calcium (mg/dL)	11.89 ± 0.68	10.64 ± 0.23	**<0.001**
Preoperative PTH (pg/mL)	238.0 ± 75.6	220.2 ± 59.5	0.56
25(OH) vitamin D (ng/mL)	22.4 ± 7.3	22.7 ± 6.4	0.75
Preoperative ALP (U/L)	152.1 ± 18.0	219.6 ± 10.5	**<0.001**
Parathyroid tumor weight (mg)	458.9 ± 58.2	588.4 ± 38.4	**<0.001**

Reference ranges: total serum calcium, [8.5–10.6] mg/dL; PTH, [18.5–88] pg/mL; ALP, [35–105] U/L; 25(OH) vitamin D, [30–100] ng/mL. Data are presented as mean ± standard deviation or number, as appropriate. PEH: postoperative early hypocalcemia; PTH: parathyroid hormone; ALP: alkaline phosphatase; “a” as Fisher’s exact test.

**Table 2:** Intraoperative and postoperative changes in PTH and calcium.

Parameter	PEH (−) (*n* = 78)	PEH (+) (*n* = 10)	*p*-Value
Intraoperative 15-min PTH decline (%)	69.9 ± 8.3	81.2 ± 4.4	<0.001
Postoperative day 1 PTH decline (%)	75.9 ± 7.1	85.5 ± 4.6	<0.001
Calcium decline at 6 h (%)	20.8 ± 4.5	27.0 ± 3.0	<0.001
Calcium decline at day 1 (%)	28.0 ± 4.2	34.2 ± 2.8	<0.001

Reference ranges: total serum calcium, [8.5–10.6] mg/dL; PTH, [18.5–88] pg/mL. Data are presented as mean ± standard deviation. PEH: postoperative early hypocalcemia; PTH: parathyroid hormone.

**Table 3:** Logistic regression analysis for predictors of postoperative early hypocalcemia.

Variable	Univariate OR (95% CI)	*p*-Value	Adjusted OR (95% CI) [Model 1: ALP]	*p* [Model 1]	Adjusted OR (95% CI) [Model 2: Parathyroid Tumor Weight]	*p* [Model 2]
15-min PTH decline (per 1% increase)	1.22		1.02	0.91	1.13	
	(1.08–1.38)	0.001	(0.78–1.31)		(0.97–1.31)	0.11
Preoperative ALP (per 10 U/L increase)	3.39		3.11	0.011	–	–
	(1.48–7.30)	0.003	(1.34–7.93)			
parathyroid tumor weight (per 10 mg increase)	1.34				1.22	
	(1.10–1.48)	<0.001	–	–	(1.10–1.48)	0.004

Reference ranges: PTH, [18.5–88] pg/mL; ALP, [35–105] U/L. Abbreviations: OR, odds ratio; CI, confidence interval; ALP, alkaline phosphatase; PTH, parathyroid hormone. ORs for ALP and parathyroid tumor weight are presented per 10-unit increase.

**Table 4:** Diagnostic performance of intraoperative 15-min PTH decline for predicting postoperative early hypocalcemia.

Parameter	Value
AUC	0.883
Optimal Cutoff (% decline)	75%
Sensitivity	100%
Specificity	74.4%
PPV	33.3%
NPV	100%
Accuracy	77.3%

Reference ranges: total serum calcium, [8.5–10.6] mg/dL; PTH, [18.5–88] pg/mL.

## Data Availability

The data are not publicly available due to ethical and privacy restrictions related to patient confidentiality; however, they may be provided by the corresponding author upon reasonable request, subject to the necessary ethical/administrative approvals.

## References

[1] Bilezikian J.P., Khan A.A., Silverberg S.J., Fuleihan G.E.-H., Marcocci C., Minisola S., Perrier N., Sitges-Serra A., Thakker R.V., Guyatt G. (2022). Evaluation and Management of Primary Hyperparathyroidism: Summary Statement and Guidelines from the Fifth International Workshop. J. Bone Miner. Res..

[2] Hargitai L., Bereuter C.M., Dunkler D., Geroldinger A., Scheuba C., Niederle B., Riss P. (2022). The value of intraoperative parathyroid hormone monitoring in patients with primary hyperparathyroidism and varying baseline parathyroid hormone levels. BJS Open.

[3] Graceffa G., Calamia S., Traina M., Contino S., Melfa G., Orlando G., Antonini R., Corigliano A., Proclamà M.P., Mazzola S. (2022). The Rome criterion: Improving intraoperative parathyroid hormone monitoring in primary hyperparathyroidism. Sci. Rep..

[4] Carsote M., Nistor C.E. (2023). Forestalling Hungry Bone Syndrome after Parathyroidectomy in Patients with Primary and Renal Hyperparathyroidism. Diagnostics.

[5] Chandran M., Bilezikian J.P., Salleh N.M., Ying H., Lau J., Lee J., Dejong M.C., Maung A.C., Parameswaran R. (2022). Hungry bone syndrome following parathyroidectomy for primary hyperparathyroidism in a developed country in the Asia Pacific: A cohort study. Osteoporos. Sarcopenia.

[6] Guillén Martínez A.J., Smilg Nicolás C., Moraleda Deleito J., Guillén Martínez S., García-Purriños García F. (2020). Risk factors and evolution of calcium and parathyroid hormone levels in hungry bone syndrome after parthyroidectomy for primary hyperparathyroidism. Factores de riesgo y evolución del calcio y hormona paratiroidea en el síndrome de hueso hambriento tras paratiroidectomía por hiperparatiroidismo primario. Endocrinol. Diabetes Y Nutr..

[7] Salman M.A., Rabiee A., Salman A.A., Youssef A., Shaaban H.E., Ftohy T., Maurice K.K., Balamoun H. (2021). Role of vitamin D supplements in prevention of hungry bone syndrome after successful parathyroidectomy for primary hyperparathyroidism: A prospective study. Scand. J. Surg..

[8] Coman A., Tarta C., Isaic A., Marian M., Olariu S., Ardelean A., Macovei-Oprescu A.M., Roland F., Pupca G.N., Latcu S. (2025). Predictors of Hungry Bone Syndrome After Parathyroidectomy in Secondary Hyperparathyroidism: A Narrative Review of Bone Turnover Biomarkers and Risk Prediction Tools. J. Clin. Med..

[9] Sun L., He X., Liu T. (2014). Preoperative serum alkaline phosphatase: A predictive factor for early hypocalcaemia following parathyroidectomy of primary hyperparathyroidism. Chin. Med. J..

[10] Tavakoli F., Yaghoubi F., Dalil D., Rezaei M. (2024). Multiple fractures due to hungry bone syndrome following parathyroidectomy: A clinical case report and review of literature. Clin. Diabetes Endocrinol..

[11] Obeidat K.A., Saadeh N.A., Msameh R., Obeidat A., Mar’ey O., Bakkar A., Manasrah Q. (2025). Predictors of Hypocalcemia Post Parathyroidectomy for Primary Hyperparathyroidism; a Single Center Study. Endocrinol. Diabetes Metab..

[12] Elfimova A.R., Eremkina A.K., Rebrova O.Y., Kovaleva E.V., Mokrysheva N.G. (2023). Prediction of the Development of Hypocalcemia in Patients with PHPT 1–3 Days after Parathyroidectomy. Endocr. Surg..

[13] Song Z., Reddy S., Wu C., Gillis A., Fazendin J., Lindeman B., Chen H. (2025). Changes in Bone Mineral Density After Parathyroidectomy in Patients With Primary Hyperparathyroidism. J. Surg. Res..

[14] Lui M.S., Clemente-Gutierrez U., Vodopivec D.M., Chang S.L., Shirali A.S., Huang B.L., Chiang Y.J., Fisher S.B., Grubbs E.G., Guise T.A. (2023). Parathyroidectomy for Normocalcemic Primary Hyperparathyroidism is Associated with Improved Bone Mineral Density Regardless of Postoperative Parathyroid Hormone Levels. World J. Surg..

[15] Sakai A., Nakao K., Kishishita S., Iwaki H. (2017). Hypocalcemia after the surgery for primary hyperparathyroidism. J. Jpn. Soc. Head Neck Surg..

[16] Fiore A., Eschlböck S., Carlen C., Lazaridis I.I., Lalos A., Droeser R., Delko T., Posabella A. (2025). Correlation between parathyroid adenoma volume and perioperative outcomes in primary hyperparathyroidism: Does the size matter?. Updates Surg..

[17] Loke S.C., Tan A.W., Dalan R., Leow M.K. (2012). Pre-operative serum alkaline phosphatase as a predictor for hypocalcemia post-parathyroid adenectomy. Int. J. Med. Sci..

[18] Hadi M., Mansouri A., Seyedyousefi S., Salehidoost R. (2025). The Effect of Preoperative Biochemical Parameters on the Development of Hungry Bone Syndrome After Surgery for Primary Hyperparathyroidism. Clin. Endocrinol..

[19] Çalişkan M., Beysel S., Kizilgül M., Özbek M., Çakal E. (2019). The effect of parathyroidectomy on bone mineral density in primary hyperparathyroidism. Turk. J. Med. Sci..

[20] Ossola P., Borasi A., Barberis A., Marola S., Ghiglione F., Pentassuglia G., Lanfranco F. (2024). Early parathyroid hormone (PTH) level as a predictor of post-surgical hypoparathyroidism. Acta Chir. Belg..

[21] Scott-Coombes D., Stechman M., Patel N., Egan R. (2024). Intraoperative parathyroid hormone assay benefits surgery for primary hyperparathyroidism when preoperative localisation is negative or not performed. Ann. R. Coll. Surg. Engl..

[22] Xu J., Kong N., Bai N., Zhang Z., Cui A., Tan S., Xu Q. (2024). Risk factors for postoperative severe hypocalcemia in patients with hyperparathyroidism: A retrospective study. BMC Endocr. Disord..

[23] Modica R., Liccardi A., Minotta R., Benevento E., Cannavale G., Colao A. (2023). Parathyroid diseases and metabolic syndrome. J. Endocrinol. Investig..

[24] Erickson L.A., Mete O., Juhlin C.C., Perren A., Gill A.J. (2022). Overview of the 2022 WHO Classification of Parathyroid Tumors. Endocr. Pathol..

